# The Effect of Formulation Excipients and Thermal Treatment on the Release Properties of Lisinopril Spheres and Tablets

**DOI:** 10.1155/2015/423615

**Published:** 2015-06-21

**Authors:** Zoriely Amador Ríos, Evone Shehata Ghaly

**Affiliations:** School of Pharmacy, University of Puerto Rico, Medical Sciences Campus, P.O. Box 365067, San Juan, PR 00936-5067, USA

## Abstract

Multiparticulate systems are used in the development of controlled release systems. The objective of this study was to determine the effect of the wax level, the type of excipient, and the exposure of the tablets to thermal treatment on drug release. Spheres from multiparticulate system with different wax levels and excipients were developed using the drug Lisinopril and compressed into tablets; these tablets were analyzed to determine the drug release. All tablets contained constant level of Lisinopril (10% w/w) and Compritol (30% and 50% w/w). Also, as a diluent, all of them contained 30% w/w Avicel and 30% w/w dibasic calcium phosphate or lactose, or 60% Avicel. Tablets compacted from spheres prepared by extruder/marumerizer and using 30% w/w lipid and 60% Avicel released 84% of drug at six hours of dissolution testing, while tablets of the same composition but prepared using 30% dibasic calcium phosphate and 30% Avicel released 101%. When the tablets were thermally treated, the drug release reduced. As the percent of lipid increased in the formulation, the drug release decreased. Compaction of tablets prepared from spheres with wax has potential for controlling the drug release.

## 1. Introduction

The development of controlled release systems has been the focus of the industrial pharmacy recently. These systems provide many advantages, such as the release of the drug for a longer period of time, fewer side effects, and more cost-effective treatments [1]. There are many factors that affect drug release from a tablet, including its composition. The type of excipient present in the formulation can affect its dissolution and its ability to compress into a tablet [2]. Also, the method used for making the tablet affects the release of the drug.

Multiparticulate systems involve the compression of many small units, which includes granules and microparticles, into one tablet; these systems are often used for controlled release [1]. Spheres are also used as one of the small units in these systems because they can be compressed into tablets, ease the distribution of the drug in the gastrointestinal tract (GIT), and can hold a high dosage of the drug; these factors contribute to the extension of drug release [3–5]. Other materials can be incorporated into the spheres in order to extend drug release, such as waxes [5].

It has been proven that it is possible to adjust drug release by integrating waxes into spheres prepared by extrusion/marumerization and compacted into tablets thereby creating a multiparticulate system for many drugs [6, 7]. The addition of the wax to the formulation retards drug release; however, melting it gives better sustained release. The melting of the wax as a result of the thermal treatment can cause its redistribution through the spheres, covering them and retarding more the drug release [7]. This is caused by the adhesion of the wax to the mucosa of the stomach, which allows the drug to stay there for a prolonged period of time [8, 9]. This mechanism can be used to manufacture medicines using different drugs to treat many conditions, like Lisinopril for cardiovascular diseases.

Lisinopril is an angiotensin-converting enzyme (ACE) inhibitor that causes vasodilation of the arteries and controls the extracellular volume [10]. This is why this drug is used for treating cardiovascular diseases, such as heart failure. For this condition, its dosage ranges from 2.5 mg to 40 mg per day [11]. The patient treated with a high dose of this medicine has to divide the recommended dosage and take its fractions through the day. With the development of a controlled release tablet for Lisinopril, the patient would only have to take his medicine once in a day, because the tablet would release the drug constantly over the period of 24 hours. This is the central objective of this study.

*Other Objectives of the Study*. They are to determine the physical and release properties of different formulations, evaluate effect of wax concentration on drug release, and assess the effect of excipient type and thermal treatment on the overall physical and release properties of Lisinopril spheres and tablets.

In order to achieve these objectives we prepared formulations containing 10% Lisinopril and 30% Compritol; 30% Avicel and 30% lactose; 30% Compritol, 30% Avicel, and 30% dibasic calcium phosphate; 30% Compritol and 60% Avicel; and 50% Compritol and 40% Avicel and all physical properties and effect of thermal treatment were investigated.

## 2. Materials and Methods

### 2.1. Materials

Four formulations were prepared and each batch was 500 g.  Table 1 shows the components of each formulation. The ingredients used were Lisinopril USP, CAS# 083915-83-7; Avicel pH101, lot number P113826449, donated by FMC Corporation, USA; dibasic calcium phosphate, lot number A045304, donated by FMC corporation, USA; lactose, lot number 8509111261, Foremost Farms, USA; and Compritol, lot number 106647, Gattefossé, Canada.

### 2.2. Preparation of Spheres

Each component was sieved trough screen number 16 (US Standard Sieve Series, Fisher Scientific Company, USA). Each batch was mixed in a V-blender (Blend Master Model B, USA) separately, adding the ingredients in the following order: first, half of the Compritol, Lisinopril, and the other half of Compritol were mixed for 5 minutes; then the rest of the excipients were added and mixed for an additional 20 minutes. To prepare the wet mass, the powder mixture was mixed in a planetary mixer (Kitchen Aid model K5SS, Hobart Corporation, USA) with the amount of distilled water shown in Table 1. This mass was passed through the extruder (model EXDS-60, LUWA Corporation, USA) to form the strands that went into the marumerizer (model Q-230, LUWA Corporation, USA). Five hundred milliliters of the extrudate was transferred to the marumerizer for 3 minutes. The spheres produced by marumerization were placed in a tray for drying at a conventional hot air oven at 37°C overnight.

### 2.3. Methods

#### 2.3.1. Standard Curve Preparation

A stock solution containing 200 mg of Lisinopril in 1000 mL of 0.1 N HCl was prepared and different sample concentrations were measured at a maximum wavelength of 209 nm with a UV spectrophotometer (DU520, Beckman Coulter, USA).

#### 2.3.2. Drug Content

For the drug content determination, three samples of 400 mg of the spheres from each batch were weighted and crushed with a mortar and pestle. Each sample was dissolved in 1000 mL of 0.1 N HCl and stirred with a magnetic stirrer (Fisher Scientific, USA) for 3 hours. Then, each sample was filtered and its absorbance was measured at a maximum wavelength of 209 nm.

Consider  *y* = *mx* − *b*, where *m* = slope from the straight line = 0.0335, *b* = intercept = 0.0433, and *r* ^2^ = 0.9988.

### 2.4. Tablet Compression and Physical Properties Determination

Four hundred milligrams of the spheres were weighted and compacted into tablets using a manual Carver pressing machine (model 4555, USA). The targeted hardness for the tablets from each formulation is shown in Table 1. The weight, the hardness, and the thickness of the tablets were measured using an analytical balance (model M-220, Denver Instruments, USA), hardness tester (Dr. Schleuniger 8M, from Pharmatron/Sotax, Switzerland), and a digital thickness gauge (number 093, Mitutoyo, USA), respectively. For the thermal treatment, the tablets were placed in an aluminum tray in the oven at 80°C for three hours. An assay for the thermally treated tablets was done.

### 2.5. Disintegration Test

The disintegration for six tablets from each batch was measured using a disintegration tester (Erweka ZT3-2, Mettler Toledo, USA) with 900 mL of distilled water at 37°C for 60 minutes.

### 2.6. Friability Test

To measure the friability of the tablets, three tablets were introduced in a friabilator (Industrial Timer Company, USA) and were processed at 100 rpm. The test was done in duplicate. The percent friability was calculated with the following equation:

(1) Percent  friability=initial  weight−final  weightinitial  weight100.

### 2.7. Dissolution Test

The dissolution of the tablets was measured in 900 mL 0.1 N HCl at 37°C in a rotary basket apparatus (model SR6, Hanson Research, USA) at a speed of 50 rpm for 6 hours. Each sample (15, 30, 45, 60, 90, 120, 180, 240, and 360 minutes) was filtered and analyzed in a UV spectrophotometer at 209 nm.

### 2.8. Data Analysis

The data obtained from the tablet thickness, hardness, and weight was analyzed using the mean and standard deviation. An ANOVA analysis was done to compare the differences of the dissolution profile of the three different formulations with different excipients. A Student *t*-test for two samples assuming equal variances was performed to compare the difference of the dissolution profile of the untreated and thermally treated tablets and the tablets with different wax levels.

## 3. Results

The objectives of these experiments were to determine the effect of the type of excipient, wax level, and thermal treatment on drug release. Spheres and tablets were successfully prepared for all formulations. When the tablets were exposed to the thermal treatment, their weight and percent friability reduced and their thickness and diameter increased (Table 2). The hardness of the tablets that contained 60% Avicel as an excipient increased when exposed to the thermal treatment; however, the hardness of the tablets that contained a combination of excipients decreased when exposed to the thermal treatment.

**Figure 1:**
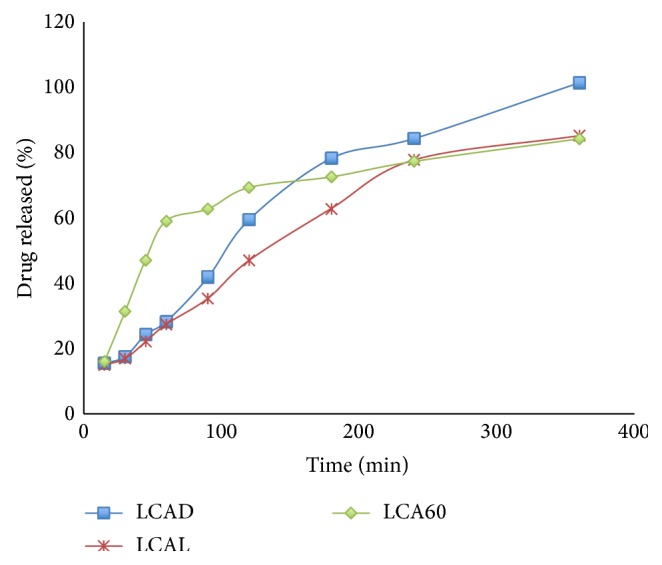
Dissolution profiles of formulations containing 10% Lisinopril, 30% Compritol, and different types of excipient without thermal treatment.

The type of diluent present in the formulation causes a variation in the dissolution profile of the drug. Tablets that gave improved drug release without thermal treatment were the ones containing 60% Avicel and 30% Compritol, which released 84% of the drug at six hours of dissolution testing. The tablets that contained 30% wax, 30% Avicel, and 30% dibasic calcium phosphate showed the poorest dissolution profile, releasing 101.4% of the drug at six hours (Figure 1). The differences between the data obtained are not highly significant, 44% of the data were significantly different (*P* < 0.05), and a Student *t*-test was done to determine which pair of excipients gave significant differences in their dissolution profiles. The differences between the dissolution profiles of the tablets that contained 60% Avicel and those prepared by using 30% Avicel and 30% lactose were significant (*P* < 0.05). These results may be due to the interruption of Avicel matrix formation in presence of other excipients.

**Figure 2:**
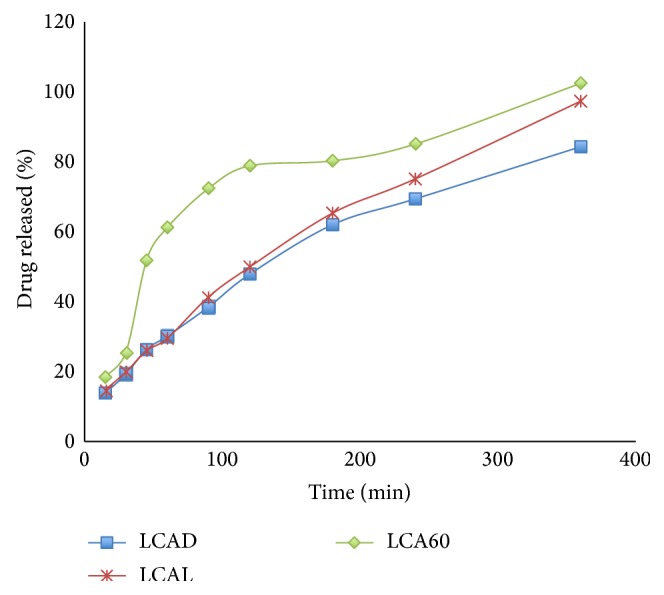
Dissolution profiles of formulations containing 10% Lisinopril, 30% Compritol, and different types of excipient with thermal treatment.

The dissolution profiles obtained for the thermally treated tablets were opposite to the ones obtained for the untreated tablets. The formulation that gave better drug release profile was the one prepared with 30% wax, 30% Avicel, and 30% dibasic calcium phosphate and thermally treated, which released 84% of drug after six hours of dissolution. The formulation that showed the poorest drug release was the one containing 60% Avicel, which released 102.4% of the drug at six hours (Figure 2). The differences between the dissolution profiles of the thermally treated tablets with different excipients were not significant; 100% of the data was not statistically significant (*P* > 0.05). The melting of the wax, which covers the spheres, masked the effect of the excipient on release of the drug; this is why the dissolution profiles for the different types of excipients, after thermal treatment, were similar.

**Figure 3:**
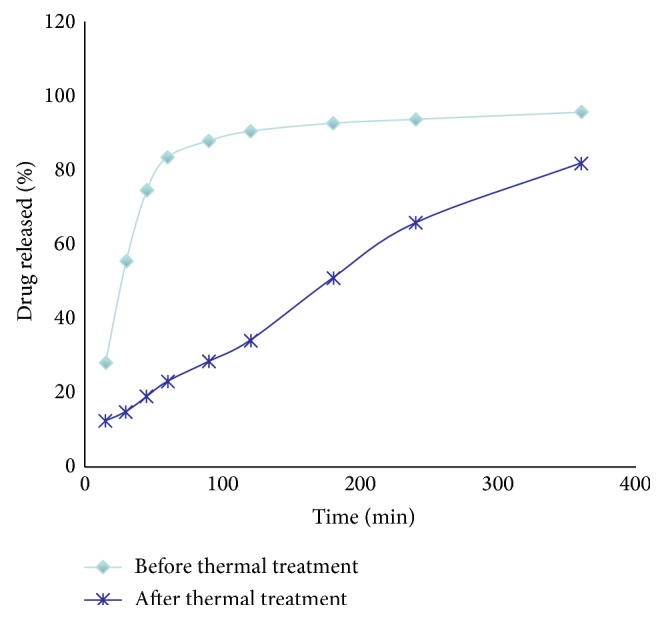
Dissolution profiles of formulations containing 10% Lisinopril, 50% Compritol, and 40% Avicel, before and after thermal treatment.

The tablets that gave better drug release were the ones that were thermally treated, which released 81% of the drug at six hours. The tablets that were not thermally treated released 95% of the drug at six hours (Figure 3). The differences between these dissolution profiles were significant; 100% of the data was statistically significant (*P* ≪ 0.05).

**Figure 4:**
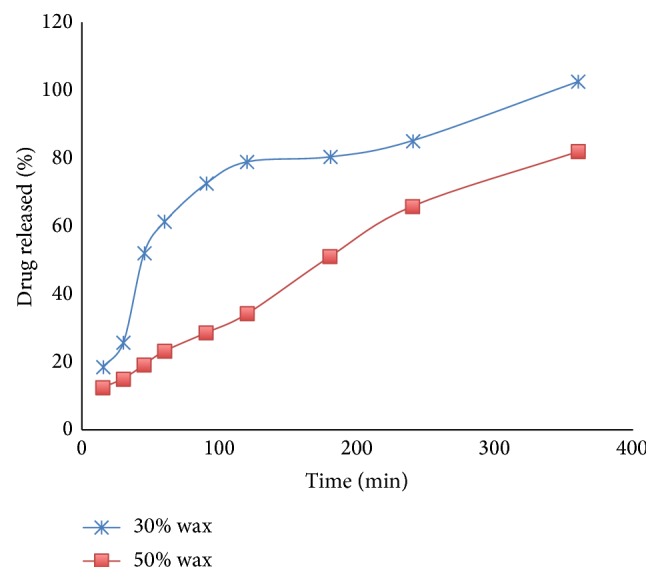
Dissolution profiles of formulations containing 10% Lisinopril, 30% Compritol, and 60% Avicel; 10% Lisinopril, 50% Compritol, and 40% Avicel.

The formulation that gave better drug release was the one that contained 50% wax, which released 81% of the drug in six hours of testing dissolution. The formulation that contained 30% wax released 102.4% of the drug in six hours (Figure 4). The differences between these dissolution profiles were not highly significant; only 44% of the data present significant differences (*P* < 0.05).

### 3.1. Kinetic Drug Release

**Figure 5:**
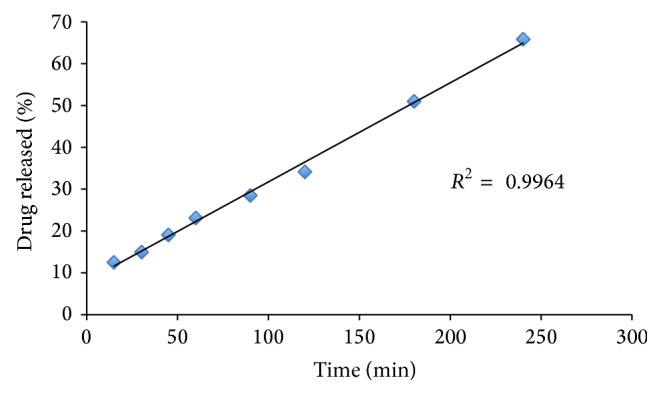
Dissolution profile of formulation containing 10% Lisinopril, 50% Compritol, and 40% Avicel.

The best formulation containing 10% drug, 50% Compritol, and 40% Avicel and thermally treated was selected to determine the drug release kinetics from the multiparticulate system.  Figure 5 shows the plot of time versus percent drug release. The plot of time versus percent drug released results in a straight line, indicating that the drug kinetic release follows zero order kinetics, where d*m*/d*t* = −*k*_0_. By plotting the diffusion model and the zero and the first order models, it was found that the zero order had the highest correlation coefficient.

## 4. Discussion of the Results

The physicochemical properties of the tablets varied when exposed to thermal treatment. The untreated tablets that contained one type of excipient gave better drug release than the ones containing a combination of excipients. The tablets with a combination of excipients gave better drug release when thermally treated, compared to the ones that contained one excipient only. The thermally treated tablets released the drug slower than the ones that were not thermally treated. Also, as the percent of wax present on the tablet increased, the drug release decreased. The physicochemical properties of the tablets changed when they were thermally treated. The weight of the tablets decreased after the thermal treatment. This could be due to the evaporation of the water added to form the wet mass. The thickness and diameter of the tablets increased when exposed to thermal treatment, which could be caused by the formation of the wax matrix when the wax melted.

The type of excipient present on the drug affects drug release. When the tablets were not thermally treated, the ones that released the drug slower were the ones that contained 60% Avicel. The thermally treated tablets that gave the better drug release were the ones that contained 30% Avicel and 30% dibasic calcium phosphate, which released 84% of the drug. These tablets released 101.4% of the drug before the thermal treatment. This means that a combination of excipients is effective in controlling the drug release of thermally treated tablets, while one excipient is effective in controlling the drug release on untreated tablets. These diluents were previously tested by Dey, Majumdar, and Rao for a control release system with thermal treatment for the drug phenylpropanolamine hydrochloride (PPA) and the same tendency was found [2].

When the tablets were exposed to thermal treatment, the drug was released slower. The thermally treated tablets released 81% of the drug, while the untreated tablets released 95% of the drug at six hours of testing dissolution. This is caused by the redistribution of the wax on top of the spheres, produced by its melting and resolidification, forming a matrix. This could be caused by the higher friability of the tablets that were not thermally treated, which prevented the complete formation of the wax matrix, releasing the drug rapidly. These results are consistent with previously reported results using different waxes and drugs [5, 7].

When the wax level varied, the drug release changed. The tablets that contained 30% wax released 102.4% of the drug, while the tablets that contained 50% wax released 81%. It is evident that the tablets with the highest percent of wax exhibited the slowest drug release. This happens because there is more wax available to form the new matrix when it melts and resolidifies. These results are consistent with previously reported results using other waxes [5].

## 5. Conclusion

Tablets prepared from spheres and with different excipients and wax levels were successfully prepared. It is possible to modify drug release of tablets by modifying the wax level and excipient type of the formulation. The dissolution profiles shown for the tablets with different excipients prove that the type of excipient affects drug release. Also, the differences on the dissolution profiles of the untreated and thermally treated tablets show that the thermal treatment slows drug release. Finally, the dissolution profiles obtained for the tablets with different wax levels demonstrate that the tablets with the highest percent of wax exhibited the slowest drug release.

## Figures and Tables

**Table 1:** Composition of all formulations and targeted hardness of the tablets.

Formula name	Lisinopril (wt%)	Compritol (wt%)	Avicel pH-101 (wt%)	Lactose (wt%)	Dibasic CaPO4 (wt%)	Distilled water (mL)	Targeted hardness (kP)
LCAD	10	30	30	0	30	370	6.5–9
LCA60	10	30	60	0	0	700	4
LCAL	10	30	30	30	0	400	7–9
LC50A40	10	50	40	0	0	560	4–5

**Table 2:** Physicochemical properties of the tablets before and after thermal treatment.

Parameters	LCAL		LCAD		LCA60		LC50A40	
	Before	After	Before	After	Before	After	Before	After
Weight (mg)	422.9 ± 6.1	404.7 ± 2.5	420.8 ± 1.5	397.1 ± 2.0	421.3 ± 1.7	420.8 ± 3.7	418.8 ± 3.9	418.7 ± 6.1
Thickness (mm)	2.99 ± 0.03	3.00 ± 0.01	2.74 ± 0.01	2.77 ± 0.02	2.98 ± 0.01	3.02 ± 0.02	3.47 ± 0.04	3.58 ± 0.07
Diameter (mm)	12.01 ± 0.03	12.06 ± 0.03	12.03 ± 0.05	12.05 ± 0.07	12.04 ± 0.05	12.14 ± 0.04	12.02 ± 0.02	12.09 ± 0.02
Hardness (kP)	8.43 ± 0.43	7.04 ± 0.38	7.35 ± 0.46	6.56 ± 0.46	2.8 ± 0.31	4.02 ± 0.34	4.09 ± 0.62	4.80 ± 0.53
Disintegration (min)	60 ± 0.00	60 ± 0.00	60 ± 0.00	60 ± 0.00	13.00 ± 3.09	31.00 ± 10.52	60 ± 0.00	47.60 ± 11.07
Friability (%)	0.05	0.00	0.06	0.00	11.77	0.07	0.00	0.00

*n* = 3; data are expressed as mean ± standard deviation. Formulation parameters were evaluated before and after thermal treatment.
